# A closer look at the international health regulations capacities in Lebanon: a mixed method study

**DOI:** 10.1186/s12913-023-10380-3

**Published:** 2024-01-11

**Authors:** Maya Hassan, Diana Jamal, Fadi El-Jardali

**Affiliations:** 1 Department of Health Management and Policy, Faculty of Health Sciences, American University of Beirut, Riad El Solh 1107, Beirut, 2022 Lebanon; 2 Knowledge to Policy (K2P) Center/WHO Collaborating Centre for Evidence-Informed Policymaking and Practice, American University of Beirut, Beirut, Lebanon; 3 Center for Systematic Reviews for Health Policy and Systems Research, American University of Beirut, Riad El-Solh, P.O.Box 11-0236, Beirut, 1107 2020 Lebanon; 4 Department of Health Research Methods, Evidence, and Impact (HE&I), McMaster University, Hamilton, Canada

**Keywords:** International Health Regulations 2005 (IHR), Capacities, Lebanon, Health system, Refugees

## Abstract

**Background:**

Lebanon ratified the International Health Regulations (IHR) (2005) in 2007, and since then, it has been facing complex political deadlocks, financial deterioration, and infectious disease emergencies. We aimed to understand the IHR capacities’ scores of Lebanon in comparison to other countries, the IHR milestones and activities in Lebanon, the challenges of maintaining the IHR capacities, the refugee crisis's impact on the development of these capacities; and the possible recommendations to support the IHR performance in Lebanon.

**Methods:**

We used a mixed-method design. The study combined the use of secondary data analysis of the 2020 State Party Self-Assessment Annual Report (SPAR) submissions and qualitative design using semi-structured interviews with key informants. Semi-structured interviews were conducted with nine key informants. The analysis of the data generated was based on inductive thematic analysis.

**Results:**

According to SPAR, Lebanon had levels of 4 out of 5 (≤ 80%) in 2020 in the prevention, detection, response, enabling functions, and operational readiness capacities, pertaining that the country was functionally capable of dealing with various events at the national and subnational levels. Lebanon scored more than its neighboring countries, Syria, and Jordan, which have similar contexts of economic crises, emergencies, and refugee waves. Despite this high level of commitment to meeting IHR capacities, the qualitative findings demonstrated several gaps in IHR performance as resource shortage, governance, and political challenges. The study also showed contradictory results regarding the impact of refugees on IHR capacities. Some key informants agreed that the Syrian crisis had a positive impact, while others suggested the opposite. Whether refugees interfere with IHR development is still an area that needs further investigation.

**Conclusion:**

The study shows that urgent interventions are needed to strengthen the implementation of the IHR capacities in Lebanon. The study recommends 1) reconsidering the weight given to IHR capacities; 2) promoting governance to strengthen IHR compliance; 3) strengthening the multisectoral coordination mechanisms; 4) reinforcing risk communication strategies constantly; 5) mobilizing and advancing human resources at the central and sub-national levels; 6) ensuring sustainable financing; 7) integrating refugees and displaced persons in IHR framework and its assessment tools; 8) acknowledging risk mapping as a pre-requisite to a successful response; and 9) strengthening research on IHR capacities in Lebanon.

**Supplementary Information:**

The online version contains supplementary material available at 10.1186/s12913-023-10380-3.

## Background

The COVID-19 pandemic brought attention back to the International Health Regulations 2005 (IHR). IHR (2005) is an instrument of international law for the World Health Organization (WHO) Member States, adopted in May 2005 and entered into force in June 2007 in response to the emergence of global outbreaks [1]. The primary purpose of the IHR (2005) is *"to prevent, protect against, control, and provide a public health response to the international spread of disease in ways that are commensurate with and restricted to public health risks and which avoid unnecessary interference with international traffic and trade"* [1]. To achieve the primary goal of protecting countries against the international spread of diseases, the IHR requires all 196 countries that ratified the regulations to maintain and develop eight capacities [2]. These public health capacities include (1) national legislation, policy, and financing; (2) coordination and national focal point communications; (3) surveillance; (4) response; (5) preparedness; (6) risk communication; (7) human resource capacity; and (8) laboratory. The IHR called for States Parties to maintain five additional core capabilities to establish the capacities at Points of Entry (PoE) and respond to zoonotic, food safety, and chemical events in addition to radiation emergencies [2]. WHO had set up components and indicators for each capacity and capability for State Parties to facilitate the monitoring and evaluation of their capacities' maintenance and identify implementation gaps (see Table 1) [3].

**Table 1:** IHR capacities and indicators

Core capacity	Indicator
C1: Legislation and Financing	C.1.1 Legislation, laws, regulations, policy, administrative requirements or other government instruments to implement the IHR
	C.1.2 Financing for the implementation of IHR capacities
	C.1.3 Financing mechanism and funds for timely response to public health emergencies
C2: IHR Coordination and National IHR Focal Point Functions	C.2.1 National IHR Focal Point functions under IHR
	C.2.2 Multisectoral IHR coordination mechanisms
C3: Zoonotic Events and the Human–animal Interface	C.3.1 Collaborative effort on activities to address zoonosis
C4: Food Safety	C.4.1 Multisectoral collaboration mechanism for food safety events
C5: Laboratory	C.5.1 Specimen referral and transport system
	C.5.2 Implementation of a laboratory biosafety and biosecurity regime
	C.5.3 Access to laboratory testing capacity for priority diseases
C6: Surveillance	C.6.1 Early warning function: indicator-and event-based surveillance
	C.6.2 Mechanism for event management (verification, risk assessment, analysis investigation)
C.7 Human Resources	C.7.1 Human resources for the implementation of IHR capacities
C.8 National Health Emergency Framework	C.8.1 Planning for emergency preparedness and response mechanism
	C.8.2 Management of health emergency response operations
	C.8.3 Emergency resource mobilization
C.9 Health Service Provision	C.9.1 Case management capacity for IHR relevant hazards
	C.9.2 Capacity for infection prevention and control and chemical and radiation decontamination
	C.9.3 Access to essential health services
C.10 Risk Communication	C.10.1 Capacity for emergency risk communications
C.11 Points of Entry	C.11.1 Core capacity requirements at all times for designated airports, ports and ground crossings
	C.11.2 Effective public health response at points of entry
C.12 Chemical Events	C.12.1 Resources for detection and alert
C.13 Radiation Emergencies	C.13.1 Capacity and resources

Under Article 54 of the IHR (2005), each State Party has to report the status of capacities' implementation annually by employing the IHR State Party Self-Assessment Annual Report (SPAR) [3, 4]. Annually, all State Parties should assess their capacities and submit the results to WHO using the SPAR tool, imposing the potential to be influenced by biases [5]. To enhance the transparency and accountability of the States Parties, the Joint External Evaluation (JEE) tool was introduced in 2015 to identify progress, ensure sustainability, find areas of strengths and weaknesses, and recommend suggestions for improving national health [6]. The scoring of SPAR and JEE tools is based on a scale scoring system. A cross-sectional study that observed the association between SPAR capacities’ scores and COVID-19 outcomes across 114 countries revealed that countries with higher IHR scores were significantly more likely to have better COVID-19 outcomes, such as a reduction in the rate of mortality and morbidity [4].

### Lebanon and the international health regulations

Lebanon, a small country of 10,452 km^2^, ratified the IHR (2005) in 2007 [7]. The country witnessed economic prosperity from the 1950s until 1975, when a devastating civil war undeniably affected the economic sector, escalating public debt and budget deficits [8]. After the end of the civil war (1975–1989), the health sector was again exposed to two Israeli ferocious aggressions: the Grapes of Wrath operation in 1996 and the 2006 war [9]. As a result of protracted instabilities, the Lebanese health system has been highly fragmented throughout history, with a strong involvement of the private sector and a widespread use of out-of-pocket payments [8]. Lebanon has been unhinged by neighboring countries' crises. Since the Israeli occupation of Palestine, the country’s rugged mountains and cities have given refuge to more than 479,000 Palestinian refugees [8]. The eruption of the Syrian crisis in 2011 has caused an estimated 1.5 million refugees to seek sanctuary in Lebanon [10]. The Lebanese health system has been challenged to respond to this humanitarian crisis, with a 30% increase in the population, that worsened the fragmentation and privatization of the health system [10, 11]. Eighty-five percent of the registered refugees live in 182 localities, where 67% of the host community lives below the poverty line [10].

Over 500,000 unregistered Syrian refugees live in informal settlements (ITSs) with poor sanitary and environmental conditions and barriers to acquiring healthcare services due to high cost and lack of accessibility [12, 13]. These poor hygiene conditions have led to outbreaks of waterborne diseases such as diarrheal diseases and hepatitis A (HAV) [14, 15]. Multiple eradicated diseases were re-introduced in Lebanon as a result of the Syrian crisis. For example, the disruption of immunization activities in Syria re-introduced measles in 2013 [16] and mumps in 2015, mainly located in the North and the Bekaa, where the highest number of Syrian refugees lived [17]. The number of tuberculosis (TB) cases has also increased in Lebanon due to treatment interruption accompanying the worsening security situation inside Syria [18]. Despite the outbreak of poliomyelitis in Syria, the leadership of the Ministry of Public Health (MOPH) succeeded in keeping Lebanon polio-free [19].

On February 21, 2020, COVID-19 hit Lebanon [20], accompanied by a severe financial crisis. Not only has Lebanon been affected by political deadlocks, financial deterioration, and infectious disease emergencies, but it has also been devastated by the third most catastrophic chemical explosion of all time after the Hiroshima and Nagasaki nuclear explosions [21]. The Beirut port explosion resulted in 600 causalities, 180 deaths, 24,600 affected migrants [22], and US$15 billion in economic losses [23]. It was reported that the blast resulted from a 2.75-kilo ton of NitroprilTM stored inappropriately due to political negligence [24]. In addition to the direct health implications, the Beirut blast released toxic gases that threatened Beirut's residents [24]. The blast implications highlighted the gap in chemical safety measures in Lebanon and the absence of appropriate preparedness and proper emergency response for chemical-related emergencies. This is ample evidence of Lebanon's vulnerability to emergencies of all types: infectious disease outbreaks, environmental changes, chemical hazards, and financial and political emergencies amplified by neighboring countries' crises.

The assessment of Lebanon's IHR capacities was done in 2016 using the JEE tool [25]. However, there have been no independent studies that expand our understanding of the Lebanese capacities to prevent, detect, and respond to public health events, especially after COVID-19 which revealed many gaps in the health system. This paper aims to gain a better understanding of (1) the IHR capacities’ scores of Lebanon in comparison to other countries; (2) the IHR milestones and activities in Lebanon; (3) the challenges of maintaining the IHR capacities; (4) the refugee crisis's impact on the development of these capacities; and (5) the possible recommendations to support the IHR performance in Lebanon.

## Methodology

### Study design

In this study, a mixed-methods research design was used. The study combined desk-based review and qualitative design using semi-structured interviews with key informants. The data from the desk-based review was used to enhance the understanding of IHR capacities’ scores, milestones, and activities in Lebanon. Whereas, the data from the qualitative interviews helped explore the challenges of maintaining the IHR capacities, the refugee crisis's impact on developing these capacities, and the possible recommendations to improve the performance of the IHR in the Lebanese context. Ethics approval to conduct this study was obtained from the Institutional Review Board at the American University of Beirut (reference #SBS-2021–0337).

### Data collection

#### Desk-based review

For the desk-based review, the authors reviewed IHR-related documents from MOPH and WHO library websites based on the following search keywords: "IHR," "health emergency," and "national health legislation." The types of documents that were found are epidemiological reports, guidelines, protocols, seminars, and commentaries. Moreover, we obtained countries' self-reported implementation percentages for 2020 as scores from the e-SPART tool to capture Lebanon's IHR capacities compared to other countries [5].

#### Qualitative interviews

We used purposive sampling to identify key informants who work with the IHR in Lebanon. Moreover, snowballing sampling was also used by asking the key informants to recommend other IHR experts. A total of twenty-six key informants, who have senior and managerial positions, were invited to participate via email from these institutions: the Lebanese MOPH (five), the WHO Lebanon country office (five), the WHO regional office for the Eastern Mediterranean (one), international organizations (six), the Ministry of Agriculture (one), the Lebanese Syndicate of Hospitals (one), the Lebanese Atomic Energy Commission (one), and independent experts (six).

Semi-structured interviews were conducted with nine (9) out of the twenty-six (26) key informants who were invited. The representation of the key informants who participated was as follows: two from the Lebanese MOPH; two from the WHO country office; one from the Ministry of Agriculture; one from the Lebanese Atomic Energy Commission; one from the Lebanese Syndicate of Hospitals; and two from international organizations. The others refused to participate due to their busy schedules or could not be reached. Initially, a consent form was sent to all key informants to participate in the interview. After obtaining their consent to participate in this study, seven interviews were conducted via Zoom or Teams based on the key informants' preferences. Two of them were conducted in the privacy of their offices. Eight of the interviews were audio-recorded, whereas meticulous note-taking replaced recording for one of the interviews based on the key informant preference. An interview guide was developed specifically for this study with open-ended questions (see online Supplemental File 1). One of the nine interviews was conducted in English, and the response notes were transcribed in English. In contrast, eight response notes were conducted in both English and Arabic, with the response notes transcribed in the language used, and then translated into English. The data was securely saved. Multiple reminders were sent out during this time of data collection.

### Data analysis

#### Desk-based review

The scoring of SPAR and JEE tools is based on a scale scoring system that can be presented in color codes and percents (see Table 2). Each indicator is scored on a 5-point ordinal scale (see Table 2). [3]. For the secondary data analysis, we analyzed the SPAR scores based on the following indices, according to a study by the WHO Health Emergency Program, Geneva, Switzerland [4]: 1) capacities to prevent; 2) capacities to detect; 3) capacities to respond; 4) enabling function; and 5) operational readiness. National scores for indicators were applied across the five health security concepts (see Table 3). Each indicator in the SPAR has a score out of 100%. Therefore, in each of the indices (prevent, detect, respond, enabling function, and operational readiness), we aggregated the scores using the following arithmetic average of indicators:

**Table 2:** IHR capacities' scores and indications

Levels	Percentages	Indication
Level 1	≤ 20%	Very little functional capacity is in place to prevent and control the risk or event
Level 2	≤ 40%	Little functional capacity available on an ad-hoc basis with the support of external resources
Level 3	≤ 60%	The country is functionally capable at the national level; however, effectiveness is low at the subnational levels
Level 4	≤ 80%	The country is functionally capable of dealing with various events at the national and subnational levels
Level 5	> 80%	The country's functional capacity is well-advanced and sustainable at all levels of health systems

**Table 3:** Capacities to prevent, detect, respond, enabling function index, operational readiness

Capacity to prevent	C3.1—collaborative effort on activities to address zoonosis
	C4.1—multisectoral collaboration mechanism for food safety events
	C9.2—capacity for infection prevention and control and chemical and radiation
	C10.1—capacity for emergency risk communications
	C11.1—core capacity requirements at all times for designated airports, ports, and ground crossing
Capacity to detect	C5.1—specimen referral and transport system
	C5.3—access to laboratory testing capacity for priority diseases
	C6.1—early warning function: indicator-based and event-based surveillance
	C6.2—mechanism for event management (verification, risk assessment, analysis investigation
Capacity to respond	C8.1—planning for emergency preparedness and response mechanism
	C8.2—management of health emergency response operations
	C8.3—emergency resource mobilization
	C9.1—case management capacity for IHR-relevant hazards
	C9.2—capacity for infection prevention and control and chemical and radiation decontamination
	C11.2—effective public health response at points of entry
Enabling function index	C1.3—financing mechanism and funds for timely response to public health emergencies
	C2.2—multisectoral IHR coordination mechanisms
	C7.1—human resources for the implementation of IHR
	C8.3—emergency resource mobilization
	C9.3—access to essential health services
Operational readiness	C1.3/ C2.2/ C3.1/ C4.1/ C5.1/ C5.3/ C6.1/ C6.2/ C6.2/ C7.1/ C8.1/ C8.2/ C8.3/ C9.1/ C9.2/ C9.3/ C10.1/ C11.1/ C11.2

$$\mathrm{Arithmetic average of indicators }= (\mathrm{C}1.3 +\mathrm{ C}2.2 +\mathrm{ C}3.1 + \cdot \cdot \cdot )/\mathrm{N}$$

These scores were compared with the average global score, the average Eastern Mediterranean regional (EMR) score, and the neighboring countries' scores of Syria and Jordan, which have similar circumstances of refugees and protracted crises.

#### Qualitative interviews

All nine (9) interviews were transcribed verbatim by the research team. Each interview was given a special number (from one to nine) for anonymity. The analysis of the data generated was based on inductive thematic analysis, which identifies patterns within qualitative data [26]. The process was done manually by using a framework suggested by Braun & Clarke [27] to analyze the qualitative data, which includes six stages: 1) familiarization with data; 2) generation of initial codes; 3) reviewing themes; 4) defining themes; 5) naming themes; and 6) producing the final report.

## Results

### Desk-based review results

#### The Lebanese IHR capacities

**Fig. 1:**
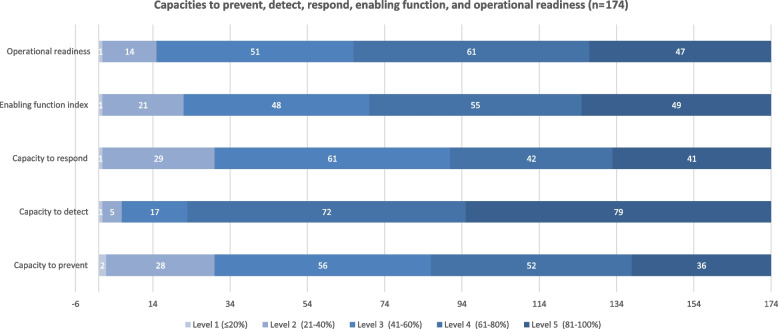
2020 capacities to prevent, detect, and respond, enabling function, and operational readiness in Member States countries

To capture the IHR capacities of Lebanon in comparison to other countries, we analyzed the 2020 SPAR submissions of 174 countries (see Fig. 1). 22 State Parties did not submit their scores in 2020; therefore, they could not be included.

##### Prevention capacity

**Fig. 2:**
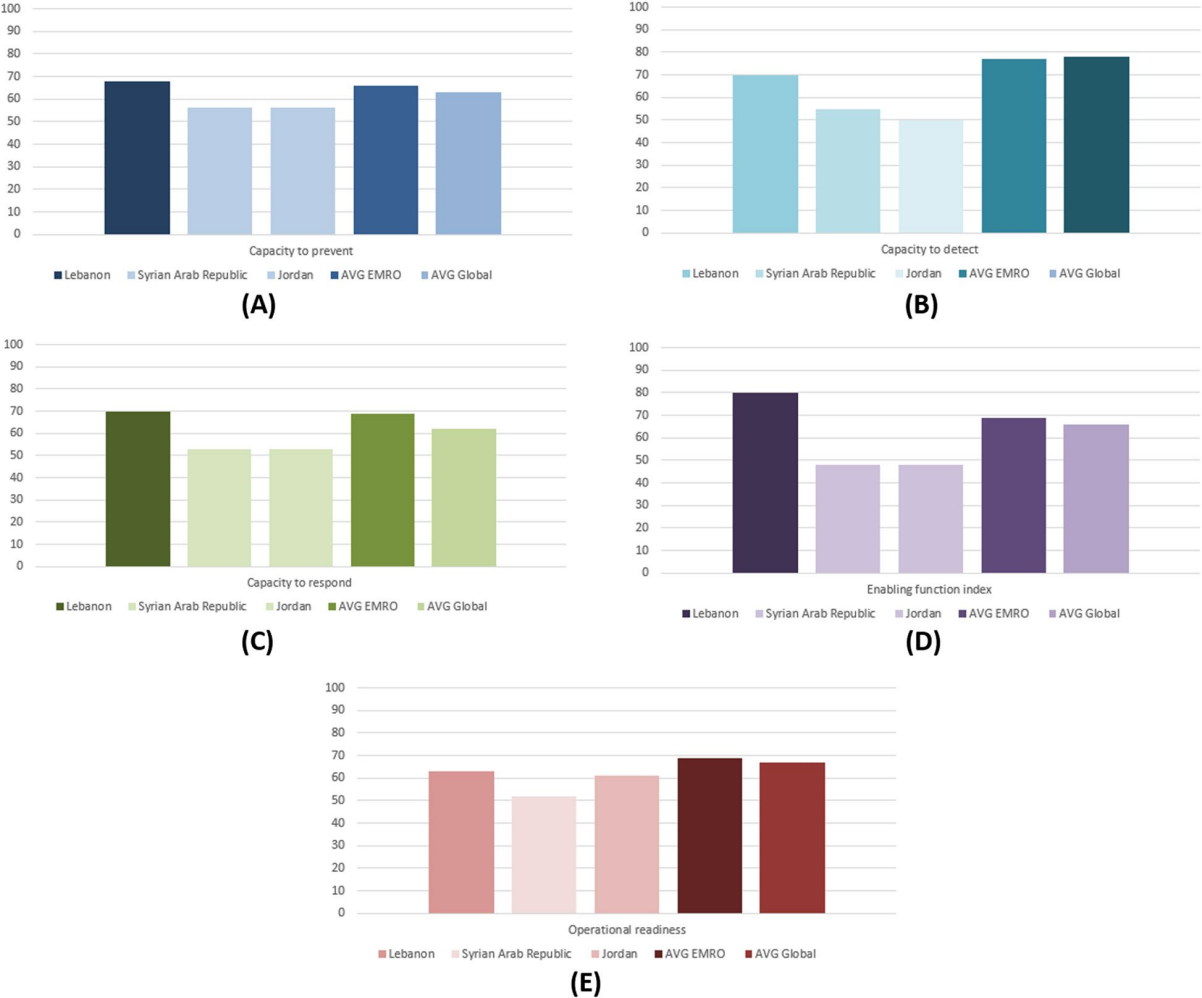
Capacities to prevent, detect, respond, enabling functions, and operational readiness in 2020 for Lebanon, Syria, Jordan, EMRO, and the global average

In 2020, Lebanon had (68%) level 4 to prevent any public health events, including infectious, food safety, chemical, and radiation emergencies. This robust prevention capacity means that Lebanon has multisectoral mechanisms, infection control capacities, risk communication practices, and requirements at the designated entry points. In this area, Lebanon scored higher than its neighboring countries, Syria and Jordan, which had level 3 (56%) in prevention capacities (see Fig. 2).

##### Detection capacity

In 2020, Lebanon had (70%) level 4 detection capacity. This indicates robust detection capacity in terms of laboratory testing resources, indicator- and event-based surveillance, and mechanisms for event management. In comparison to Syria and Jordan, Lebanon is doing more robust work in the detection area; however, Lebanon scored less than the EMRO and the global average (see Fig. 2).

##### Respond capacity

Lebanon had a robust capacity to respond to public health events, scoring (70%) level 4. This is more than its neighboring countries, Syria and Jordan, and more than the global and regional average.

##### Enabling function

In terms of enabling function, or the levels of resources and collaboration to prevent, detect, and respond to an emergency, Lebanon had level 4 (80%) of enabling function. Lebanon also highly exceeded Syria and Jordan in this area. Both countries had level 3 (48%) due to a resource shortage and collaboration to prevent, detect, and respond to an event.

##### Operational readiness capacity

In 2020, Lebanon had 63% (level 4) of operational readiness capacity, meaning that it has a moderate to high level of readiness to respond to emergencies effectively and efficiently. Similar to the above capacities, Lebanon is doing better work than the neighboring countries of Syria and Jordan in this area.

#### Milestones and activities of the IHR in Lebanon

**Fig. 3:**
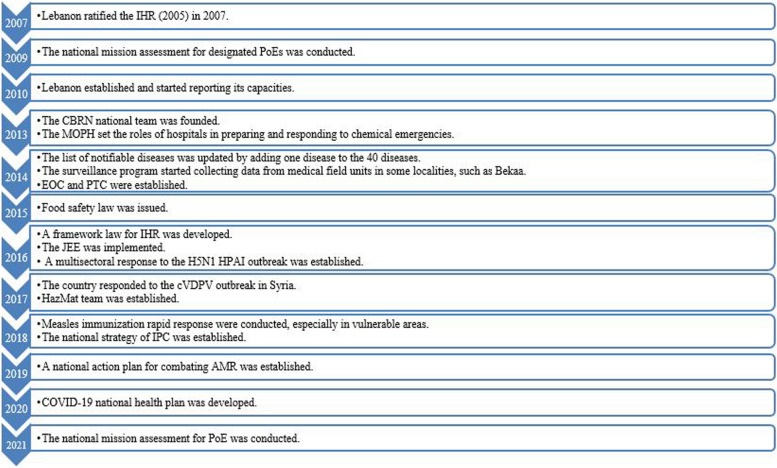
Timeline of IHR (2005) milestones and activities in Lebanon

The findings are presented here within two-time intervals, as recommended by IHR key informants: 2009–2014 and 2015–2022 (see Fig. 3). We also categorized the milestones by capacity.

##### 2009 – 2014 milestones and activities

Lebanon ratified the IHR (2005) in 2007 [28]. In 2010, it was one of the countries to establish and start reporting its capacities. In the area of legislation and financing, Lebanon has assigned a national focal point and an alternative focal point. In terms of surveillance, the list of notifiable diseases was updated in 2014 by adding one disease to the 40 diseases [28]. An event-based surveillance system that uses community, social media, and a hotline was also established. In 2014, with the increasing risk of communicable disease transmission due to the poor living conditions of refugees, the surveillance program started collecting data from medical field units in some localities, such as Bekaa [29]. In the area of early warning and response (EWARS), an emergency operation center (EOC) and a professional training center (PTC) were established in 2014. The EOC was established to be used in cases of public health events, whether chemical, radio-nuclear, or natural disasters [30]. In 2014, many activities were implemented in the EWARS area, such as developing a response plan to respond to the 1292 cases of cutaneous leishmaniasis (CL) among Syrian refugees [31], launching the national polio immunization campaign [32], strengthening cholera preparedness and response plan, particularly in the ITSs [19], and launching the severe acute respiratory infections (SARI) program with the enrollment of 10 sentinel sites [33].

In the areas of chemical events and radiation emergencies, the chemical, biological, radio-nuclear, and nuclear national (CBRN) team was founded in November 2013, decision 179/2013 [34]. It includes representatives from the Lebanese Atomic Energy Commission (LAEC), the Lebanese Army, the General Security, the State Security, the Ministry of Foreign Affairs, the Internal Security Forces, the Presidency of the Council of Ministers, the Civil Defense, the Lebanese Customs Administration, the Ministry of Environment, the Ministry of Industry, the Ministry of Agriculture, and the MOPH [34]. In 2013, the MOPH set the roles of hospitals in preparing for and responding to chemical emergencies. This includes updating the preparedness plan for every hospital, healthcare workers' training, and ensuring medication supplies and personal protective equipment (PPE). Moreover, MOPH set 17 hospitals as reference hospitals for chemical emergencies [35–37]. In May 2014, training on the different phases of the emergency cycle was conducted on CBRN hazards in nineteen hospitals [38]. One major milestone at the PoE was implementing the national mission assessment for capacities at the designated PoE in 2009.

##### 2015 - 2022 milestones and activities

One milestone is the implementation of the Joint External Evaluation (JEE) in 2016, where Lebanon scored relatively well (4/5) [25]. In the area of legislation and financing, Lebanon drafted an IHR framework law and corresponding sectoral laws in 2016. Although the law was not passed, the framework included amendments to laws and legislation related to IHR implementation in several ministries and entities: the MOPH, the Ministry of Agriculture, the Ministry of Environment, the Ministry of Public Works and Transport, and the Ministry of Economy and Trade. In the area of the national emergency framework, EWARS was strengthened through several measures, including updating operating procedures for 43 selected diseases; distributing surveillance guidelines to hospitals and clinics; training 133 personnel on standard operating surveillance and response procedures; training health educators on school-based surveillance; and establishing 8 negative pressure rooms [19]. Since 2015, Lebanon has responded to multiple outbreaks, including the 2016 H5N1 HPAI outbreak in Baalbek, with the involvement of the MOPH, the Ministry of Agriculture, the Lebanese Armed Forces, the Interior Security Forces, and the High Relief Commission [39, 40]; the 2017 cVDPV outbreak in Syria, through the national surveillance of AFP cases and the acceleration of routine immunization and continuous vaccination at all borders [41]; and the measles immunization rapid response in 2018 [42]. After the emergence of COVID-19, Lebanon developed a national health plan to scale up preparedness and response capacities through organizing training for health and non-health staff at PoE, establishing referral protocols from PoEs to health facilities, and providing technical support to specific PoE [43, 44].

In terms of food safety, a law was issued and training sessions for 170 public health inspectors were conducted in 2015 [45]. For chemical events and radiation emergencies, a medical hazards management team (HazMat) was established in 2017 and trained on CBRN response [25]. In the area of PoE, a national mission assessment was conducted in 2021 to evaluate capacity requirements for 20 designated PoE, including airports, ground crossings, and ports. It was noticed that the PoE lacks the required equipment and procedures under IHR. Quarantine-required measures are absent in most PoE and waiting areas are absent in almost all PoE. According to the assessment, many activities were done between 2009 and 2021, including assigning two rooms as quarantine centers in Beirut Rafic Hariri airport, introducing special exits for ill travelers, and deploying health authorities to all land crossings.

In the area of health service provision, the IPC national strategy was established in 2018 [46]. The plan included establishing policies and procedures, ensuring staff education, reducing healthcare-associated infections, cleaning and sterilizing medical devices, managing wastes properly, ensuring sharp safety, providing food safety, complying with hand hygiene, maintaining a clean physical environment, monitoring quality indicators, and training hospitals on the use of WHONET software to report Lebanon's antimicrobial resistance (AMR) data through the global AMR surveillance system (GLASS) [47–49].

### Qualitative results

The findings revealed the following themes: IHR challenges, IHR strengths, IHR and refugees, and recommendations to support the performance of IHR in the context of Lebanon. The Summary of the qualitative analysis is presented in online Supplemental File 2.

#### Challenges facing the IHR in the Lebanese context

Five main themes emerged concerning IHR challenges in the Lebanese context: resource shortage, political challenges, governance challenges, and gaps in IHR knowledge. Almost all key informants agreed that the main challenge nowadays is the economic crisis that deprives the country of its human and financial resources. All key informants asserted that the shortage in resources, whether human, financial, supplies, power, or information systems, enormously affects the implementation of IHR and its capacities in Lebanon. All key informants reported the human resources deficit, especially in the non-centralized districts and the portal areas, as a major barrier to conducting IHR activities, mainly due to the alarming migration rate of human resources from Lebanon after the latest crisis. As stated by one of the key informants in MOPH:

*“Today, I need electricity… I need Internet… give me electricity and Internet… give me a team... My team is migrating… I lost many staff from my team… some migrated to Chili… some to the USA… to France… to Egypt… to the UAE… the team of the Ministry is migrating one after another… I need my team to stay with me… I cannot force them to stay… If you train the staff, they will go… they will travel anyway… you and I cannot fix this problem…”*

Financial resources shortage was also reported as a primary challenge by almost all of the participants. It was disclosed that IHR funding relies solely on WHO funds, without any private entities' investments. This has created an actual burden, especially with the devaluation of the currency in Lebanon. One of the key informants reported:

*“I think the main challenge to doing anything under IHR is actually the budget because usually, there is a trust fund from the Ministry of health with WHO to work on IHR... So now, with the devaluation, it is worth nothing... And with all the competing priorities, there is no real funding to implement anything or to strengthen any of the aspects under IHR…For example, the health clinic in the port of Beirut was destroyed, and it is still… the staff work in a destroyed place, and many stuff fell on their heads…”*

Moreover, this financial deficit has led to a shortage of supplies and essential kits, affecting some of the IHR's main activities in the active surveillance area. As one of the key informants emphasized:

*"Recently, when avian influenza emerged in occupied Palestine… We demanded from all the points of entry to talk with animal breeders to take the appropriate measures with the chickens… we took samples only when there was a suffocation case because now there are shortages in staff, finance, and kits. So I prefer to leave the kits to the real threats, not the suspected ones..."*

In addition to the financial deficit, the power deficit in Lebanon is playing a fundamental role in hampering key activities and tasks. For instance, fuel shortage has a major negative impact on data collection and reporting under IHR capacities. As a consequence of fuel shortage, there is an undersupply of Internet and electricity. Information systems are other types of resources that have challenged the IHR performance in Lebanon. Although these systems are essential stones for the surveillance system, many obstacles are facing the installation of such systems:

*“In the hospitals, we have challenges in reporting… sometimes, they ask us to have computerized systems, and to have software that speaks to each other… this requires from the hospital to have software, equipment, and the trained staff…”*

Six key informants acknowledged the political context in Lebanon and its features of political pluralism, mismanagement, and corruption as the main challenge. This complicated political context has led to the disruption of some activities under the IHR. For instance, it was reported that it is problematic to conduct investigations under IHR in several areas in Lebanon due to the control of specific political cleavages. Moreover, as reported by one key informant, the political context in Lebanon has resulted in suspending the activation of laws under IHR:

*“The legislations have been developed. It took three years to develop a draft with the sectoral laws, which means each institution or Ministry. They have to change some of their mandates so they do not overlap to become accountable, and they have a specific role in IHR. This did not pass. This is, of course, related to the political cleavages and all the context in the country…”*

One consequence of this political context is the public's mistrust of the government and its laws and legislation. Public incompliance poses a major challenge in responding to public health emergencies. As one of the key informants said:

*"The most important thing is the public compliance… I do not know if this is an awareness issue or mistrust in the public sector. When the government issues one legislation regarding the pandemic or IHR… people did not have trust… this was a challenge…."*

Five key informants believe that the governance of the IHR is the main challenge in the context of Lebanon. The absence of an accountability framework has led to issues of commitment, credibility, miscoordination mechanisms between some entities, and lack of transparency in reporting. This is evident in the absence of a central public health laboratory with a clear mandate in the country. As one of the key informants mentioned:

*"Each component has its own challenges, actually, but if you look at it overall, it is an issue of governance; it is the way the government deals with IHR. It is the overlapping mandates of the concerned authorities. It is also the commitment actually at a higher level for the preparedness part…you do not have any things quick. It is not the expertise that is missing; it is not the infrastructure that is missing. It is really the governance, the commitment, the willingness to coordinate, to establish the mechanisms once and for all, to have it centralized under one umbrella, which should be the IHR central committee that does not exist."*

Another key informant mentioned that the overlapping mandates in some areas are causing a conflict of power. This was evident in the food safety area:

*"Food safety is a vast domain, and you can say it is an area of power conflict between different ministries… In all over the world, the head of the food safety area is the Ministry of Agriculture…Here it involves the Ministry of Economics, the Ministry of Public Health, the Ministry of Industry... other NGOs. This is causing a conflict."*

Two of the key informants working outside WHO and MOPH revealed a gap in IHR knowledge in the non-governmental organizations (NGOs) sector. Additionally, one of the key informants asserted that there is an IHR awareness issue among non-health staff at the PoE.

##### IHR strengths in Lebanon

Four main themes emerged in this area: constant collaboration, high-level capacity, IHR awareness, and preparedness for the following emergencies. Although three key informants asserted a gap in collaboration, five out of nine respondents reported that several agencies are committed. They are keen to have constant collaboration under the IHR. Another respondent mentioned that the CBRN team is clear evidence of the multisectoral collaboration since it involves representatives from several entities. Other key informants asserted that the constant communication between MOPH and WHO is one of the main strengths of IHR implementation in the Lebanese context. In addition to collaboration between entities inside Lebanon, collaboration with other entities outside the country plays a major role in resource pooling and sharing. For instance:

*"The Ministry of Agriculture has the Lebanese Agricultural Research Institute, which is under the supervision of the Minister… we do all of our lab investigations there… in case we need confirmation… For example, when we did the H5N1 lab testing, we did it there. And the result was positive… After that, we sent it to Italy through the OIE, and we got the same result… just for confirmation… And if we do not have the lab test here, we send it to a reference lab outside the country."*

Four key informants reported that Lebanon has a high level of capacity. Despite the shortage in the density of human resources, there are trained and equipped experts who can develop and organize contingency plans to face and respond rapidly to any public health events. Moreover, in terms of capacities, only a few key informants asserted that Lebanon has an excellent evidence-based surveillance system in addition to excellent laboratory capacity. For instance:

*"What has been done for the COVID in Lebanon was actually very, very good. An example is: Lebanon was the first country in the region to be able to take in patients with COVID at the early beginnings. Why? Because under the IHR, we had at RHUH*^1^* four isolation rooms very equipped with PPEs, ABC and d level PPEs, we had trained nurses, we had trained medical doctors on IPC and on isolation etc., but what was the preparedness component then came COVID and then they used that. So this was excellent."*

Another strength that was reported twice is the MOPH's awareness of IHR components. Additionally, Lebanon's multiple crises and emergencies made the country more resilient and prepared for future emergencies. One of the key informants reported:

*"I can tell you that we will have a more rapid response if another pandemic emerges because we are prepared now…For instance, the fragmentation of data took some time to be fixed, but now we are better in terms of response."*

##### IHR and refugees

When key informants were asked about the impact of the refugee crisis on the development of IHR's capacities in Lebanon, three main themes emerged: positive, negative, and neutral perspectives. One key informant believes that the Syrian refugee crisis has had a positive impact since it revealed the weaknesses in the Lebanese health system. Another key informant asserted that the Syrian refugee crisis has led to increasing humanitarian funds and strengthening the coordination mechanisms between different entities:

*“I do not think that refugees would cause a country to have weaker capacities to intervene to certain emergencies… whether IHR capacities… either the country knows how to adapt… how to basically serve all of its residents… or it doesn't… we need to look that a lot of funds came because of the refugees… this is very important point.. The Syrian crisis itself created a coordination mechanism between the partners … between the government and the UN agencies….these coordination mechanisms helped us to have coordination mechanisms in COVID…”*

Three key informants have neutral perspectives. They believe that refugees' presence does not interfere with IHR development. As one of the key informants mentioned:

*“They do not interfere in the core capacities development because the surveillance is there. It is expanded automated; the preparedness is there… What is probably affecting the capacity of the country is not the refugees; it is the economic situation in the country…”*

Moreover, four key informants stated that the Syrian refugee crisis increased the prevalence of infectious diseases and the risk of introducing eradicated diseases due to the unhealthy environment refugees have been living in and the gap in awareness messages targeted toward refugees. For instance:

*“They suffer from other diseases… the Leishmaniasis cases have increased… other diseases started to be introduced… they have a profile of infectious diseases…”*

##### Recommendations to support the IHR in the context of Lebanon

Four main themes emerged: coordination strengthening, risk communication and community engagement (RCCE) strategies, resource investment and strengthening, and risk mapping. Almost all key informants agreed that to strengthen the implementation of IHR capacities in Lebanon, multisectoral coordination mechanisms between all concerned entities should be strengthened. It must be emphasized that the IHR is not the responsibility of the MOPH alone; it is a multisectoral responsibility. For instance:

*“… I think the first lesson to learn is that ownership of IHR should be multi-disciplinary, multisectoral, and it should be very high-level decision-making for IHR. It cannot be thrown on the Ministry of public health alone because quarantining an area, for example, which is a response issue, is not the mandate of the Ministry of health. It needs the abidance of the Ministry of the interior and the armed forces. You name it. So I think this is important to have a very senior national committee that takes the decision…”*

Moreover, it was asserted that continuous coordination, presented by more meetings and communication channels, not only in the presence of certain threats, is required. There is also a need to involve NGOs in the process. For example, one health coordinator in an NGO emphasized the need to involve other NGOs under the IHR mandate:

*“I would say I have worked in the humanitarian sector for 6 years, and in none of the meetings with WHO and MOPH, I learned about the IHR. We should know what IHR is, what the updates are, the plans; in this way, we can help in finding funds and assigning a budget and financial requirements, so we can help…”*

Four key informants asserted the necessity of strengthening RCCE strategies, especially those targeted toward vulnerable populations in decentralized localities. Another recommendation reported by four key informants is strengthening investment in resources, whether financial or human. As asserted by key informants, increasing the financial resources could be done through shifting resources towards IHR, fund pooling, and fundraising. Strengthening human resources, whether through continuous training or simulation exercises, was frequently mentioned. One key informant emphasized the need to train human resources, especially in the portal areas, where IHR awareness is at the minimum level:

*“There is a gap in the knowledge… not centrally, but more for the staff at the portals… I can tell you, for instance, we worked with the CDC portal management team on the health capacities on the portals… after the assessment, we noticed that other authorities: the customs… the general security, they have no clue that they had a role if they see someone sick to refer them not to have them pass… If someone has a fever… so, we had to train the non-health staff to know that they have a role in health…”*

Moreover, one key informant emphasized the need to deploy human resources for the EOC to benefit from its capacities:

*“We have public health emergency operation center… we have one in Lebanon, and it is good… it is in the RHUH*^2^* under the supervision of the MOPH… you need to have people who are recruited for this emergency room…you do not need to have staff who go to this emergency room when there is an emergency… you have to have trained staff who know their roles and who are recruited specifically for this PHEOC…”*

Finally, risk mapping was mentioned by two key informants as a recommendation to strengthen IHR performance. This will help identify and prioritize the infectious, chemical, and radiation emergencies that may happen in Lebanon. For instance:

*“It is important to have risk mapping… this is still not achieved in Lebanon…it is very important to highlight the importance of risk mapping… it tells us which emergencies may happen… whether infectious… chemical or other… this is a lesson that we should learn from the Beirut blast to have mapping of all risks…”*

## Discussion

This is the first study to provide mixed-method findings on the status of IHR capacities in Lebanon. It delivered results of high importance, especially after the emergence of COVID-19. Lebanon was one of the 65 (33%) State Parties that met the minimum core capacity standards in the meeting of the IHR Review Committee in November 2014, while the other 81 States Parties had requested a two-year extension and 48 had not communicated their intentions to the WHO [50]. Despite this high level of commitment to meeting the IHR capacities, our findings demonstrated several gaps in the IHR performance due to the perpetual challenges Lebanon has been facing on the economic, political, and social levels [51].

This study introduced a new approach to understanding Lebanon's IHR capacities by analyzing e-SPAR scores and complementing them with an in-depth knowledge of IHR experts. We only analyzed the 2020 capacities' scores as a result of multiple revisions in the e-SPAR tool, which made it impossible to study the trend of scores over time [3]. We categorized the scores into five health security indices: prevent, detect, respond, enabling function, and operational readiness, based on a study done by the WHO Health Emergency Program in Geneva [4]. Lebanon had levels of 4 (≤ 80%) on the five indices. The country scored more than its neighboring countries, Syria and Jordan, which have similar contexts of economic crises, emergencies, and refugee waves. These scores indicate that the country has adequate resources and national plans to prevent, detect, and respond to any future emergencies. This contradicts the qualitative findings, thus posing a concern about those indices' ability to capture the actual performance of the IHR capacities. This contradiction may arise from the fact that all indicators are given the same weight. For instance, in the prevention capacity, more work should be done on the entry points, zoonotic, and food safety indicators, where the country scored less than other indicators in the prevention area. Additionally, to enhance its detection capacities, Lebanon has to work more on the laboratory specimen referral and transport system indicator, in which it scored less than the other indicators. Although Lebanon exceeded its neighboring countries' scores in terms of enabling function and operational readiness to respond to future public health emergencies, it is still not making efforts in many indicators, such as human resources capacities and multisectoral collaboration under the IHR.

Our paper investigated the milestones that Lebanon has executed since it ratified the IHR in 2007. Although we cannot capture all milestones due to data limitations, it was evident that the country has been advancing its capacities (Fig. 3). Despite all the efforts that the country has made to strengthen its IHR capacities, challenges still exist, as reported by the key informants. For instance, many gaps exist in the legislation area, most notably the failure to pass the framework law for IHR implementation that aims to recognize the IHR framework as domestic law and incorporate it within the concerned ministries and entities, as many countries did: France, Finland, Syria, Sweden, and Australia [52]. This absence of an accountability framework may be attributed to bureaucratic hurdles and vested interests, the main characteristics of governance in Lebanon, leading to miscoordination mechanisms and a lack of commitment among some concerned entities. According to the IHR Review Committee on COVID-19, effective IHR implementation requires political commitment nationally and internationally [53].

Lebanon has made some progress in its coordination capacity; however, the multisectoral IHR coordination mechanisms are still not fully functional. A definition of multisectoral collaboration should be revisited to identify the key entities and their responsibilities. Multisectoral coordination was emphasized in a study conducted on IHR in Yemen, which recommended improving the alignment of international non-governmental organizations programs with government health programs and aligning both towards better implementation of the IHR [54].

This study also shows that the country has been working hard on the national emergency framework by strengthening the EWARS [35], through developing operating procedures, contingency plans, and surveillance guidelines for many diseases, including zoonotic ones, in addition to conducting simulation exercises and training. However, the shortage of human resources is impeding such efforts. This is inevitable in a country like Lebanon, as the World Bank warned that “brain drain” or the alarming migration of qualified people is an “increasingly desperate option” as a result of one of the most severe crises in the world [55]. The Lebanese crisis has not only affected the human resources area but also impacted the surveillance capacity, a critical pillar in IHR and an area where the country has been making much effort to strengthen and produce one strong epidemiological surveillance program. The negative impact of the Lebanese crisis was emphasized by key informants who reported that the deficit in the infrastructure of electricity and the Internet is impacting the daily routine of indicators and event-based surveillance systems. For Lebanon to have a robust and resilient surveillance system that can detect all public health risks, infrastructure, and resources should be in place. The laboratory is another essential area that Lebanon was doing fair in strengthening it. One impressive strength here is Lebanon's commitment to the WHO recommendation of pooling international laboratory resources through collaborating centers at the local, national, regional, and international levels [3]. However, the main challenge in the case of Lebanon is the absence of a central laboratory [25], while its neighboring country Jordan possesses such a laboratory [56].

The qualitative findings in our study revealed that RCCE measures are being implemented on an ad hoc basis in Lebanon. More investment in RCCE measures is necessary while ensuring the inclusion of vulnerable populations such as Syrian, Palestinian, and Iraqi refugees and migrant workers, who experience marginalization amidst the absence of equitable social protection schemes [57].

Lebanon, as stated by the IHR, embraced an “all-hazard” strategy. For this, it acknowledged zoonotic diseases, radio-nuclear, and chemical emergencies as actual emergencies. This was evident in the foundation of the CBRN national team in 2013 and the HazMat teams in 2017 [25, 34]. This does not diminish the need to strengthen the financial and human resources to detect and respond to chemical and radiation emergencies, especially after the Beirut blast implications that highlighted the gap in chemical safety measures in Lebanon and the absence of appropriate preparedness and proper emergency response for chemical-related emergencies [24]. This was also highlighted in the JEE report, which emphasized the need for a national strategic plan for chemicals, reflecting the needed workforce and financial resources [25]. At the human-animal interface, Lebanon was able to prepare contingency plans for many zoonotic diseases, such as avian influenza. However, the shortage of kits, materials, and staff at some localities is hampering the ability to conduct ongoing investigations. Other suspected hazards are foodborne diseases. The reported gap here is the overlapping mandates between different entities, with the involvement of the Ministries of Health, Industry, Economy, Trade, Agriculture, and producers and consumers, causing a conflict of power [25].

The former gap in coordination is also present at the PoE in Lebanon. The 2021 PoE assessment highlighted the deficiency in the required equipment and procedures under IHR at the ground crossings and ports in Lebanon due to the absence of the non-health authorities' commitment. This challenge was asserted in this qualitative study, highlighting the need to have more involvement and commitment from the non-health authorities, such as Customs and General Security. The Lebanese political context of pluralism and mismanagement hinders any progress, especially with the importance of entry points in trade and economic profits, placing it as an area of corruption.

To our knowledge, no study before has investigated the impact that refugees impose on IHR (2005) capacities in a country. Key informants reported that the refugee crisis increased the prevalence of infectious diseases. This was evident between 2013 and 2019 for HAV, CL, mumps, and measles [58]. One interesting finding in our paper is the positive impact that refugees had on IHR performance according to some key informants; however, this could not be coupled with quantitative evidence from the e-SPAR tool. The refugee influx required Lebanon to establish coordination mechanisms between many entities, including ministries, the private sector, NGOs, and UN agencies. These mechanisms were used and strengthen the coordination mechanisms in the area of IHR. Whether refugees interfere with IHR development is still an area that needs further investigation. However, it is clear that the IHR capacities and its monitoring tools, such as e-SPAR and JEE, have not addressed whether refugees, displaced persons, and migrants should be integrated into the national health prevention, detection, and response approach in addition to the need to support the countries that host refugees.

To our knowledge, this is the first study to explore the performance of IHR in the context of Lebanon since 2016, when JEE was conducted. Another strength of our paper is that it uses a mixed-method design to expand the understanding of IHR status in Lebanon. Moreover, we interviewed key informants from different entities and institutions. Our paper also has some limitations. The e-SPAR tool was revised multiple times since its establishment, which hampered the ability to do detailed quantitative analysis of trends over time. Another limitation is that the number of interviews was relatively few (*n* = 9) because of the difficulty of reaching IHR experts due to their busy schedules. Interviewing healthcare professionals, field workers or patients would have also enriched the paper.

## Implications for policy and research

Many policy and research implications have arisen from the desk-based review and qualitative analysis of this study. We believe that Lebanon’s experience in implementing IHR (2005) capacities is unique and useful for countries facing the same norms of new and emerging political, social, and economic crises.

### Reconsidering the weight given to IHR (2005) capacities when assessing them

Despite the significant challenges Lebanon is facing, the findings of this study highlighted that Lebanon is doing relatively well in preventing, detecting, and responding to public health emergencies. This poses a concern about whether all the capacities should be given the same weight when assessing them. The Delphi technique could be used with IHR National Focal Points to prioritize the most critical indicators [59].

### Promoting governance to strengthen IHR compliance

With the compounded crises Lebanon has been assailed by, good governance is a prerequisite to strengthen IHR compliance. The main principle of promoting governance is establishing an accountability framework to ensure participation and transparency with open information systems [60]. Despite the current political instability in the country, adopting the law is detrimental to establishing an accountability framework for all the concerned parties. This could be done by developing advocacy plans to mobilize political commitment, taking advantage of windows of opportunities such as the COVID-19 pandemic that had an enormous impact on the country's social, economic, and political spheres. Without good governance, multisectoral collaboration cannot be effectively achieved.

### Strengthening multisectoral coordination mechanisms

While solid participation by the health and agriculture sector is common in Lebanon, it is not solid in the non-health sector. Nowadays, the increasing public health risks, such as biological, chemical, and radio-nuclear, highlight the responsibility of the non-health sectors in public health risk prevention and control. Cross-sectoral collaboration is essential, and the sectors include but are not restricted to, animal and human health, chemical and radiation safety, the Army, Defense, the Internal Security Forces, General and State Security, the Customs administration, finance, transport, foreign affairs, and the Media. Establishing communication channels as electronic platforms is crucial to facilitating the efficient and timely transmission of information [61]. These channels would ensure constant communication instead of the existing ad-hoc networks. Moreover, partnerships with civil society organizations (CSO) and dedicating significant effort to raising awareness among them about IHR (2005) could also serve as an advocacy approach to ensure political commitment, especially with their success stories in influencing the policy-making process in the country [62]. CSOs in Lebanon are known for their role in monitoring the government and holding stakeholders to account. Additionally, technical cooperation with international organizations, especially those providing services to refugees and displaced persons, should be enhanced to avoid duplication of resources and efforts.

### Reinforcing risk communication strategies constantly

This study showed the gap in risk communication strategies in Lebanon that is being done on an ad-hoc basis. The findings of this study established the need to conduct constant RCCE strategies, not only during public health emergencies.

### Mobilizing and advancing the capacities of human resources at the central and sub-national levels

After the recent economic crisis, Lebanon started suffering a dangerous depletion of human capital. Therefore, it is essential to begin by identifying gaps in the workforce in terms of localities and competency to ensure that training and resources are targeting the needs [61]. Constant training and simulation exercises are required, especially with the high staff turnover.

### Ensuring sustainable financing

In Lebanon, IHR development relies on WHO and funds allocated by MOPH to establish health units at the PoEs. Due to the economic crisis, the currency devaluation, and the competing priorities, there is a huge need to allocate sustainable funding for IHR development. This could be done in partnership between the government, international organizations, and non-State actors [61]. Article 44 of IHR (2005) affirmed that States Parties should collaborate to mobilize financial resources [1]. Therefore, increasing WHO Member States' contributions to countries facing emerging challenges is a logical funding resource.

### Integrating refugees and displaced persons in the IHR (2005) framework and its assessment tools

The desk-based review revealed that the IHR framework and its assessment tools, such as JEE and e-SPAR, did not address how refugees and displaced populations are integrated into the country's prevention, detection, and response approach to public health emergencies. WHO leadership should take a fresh look at IHR implementation in countries with large displaced populations to discuss with its partners, such as the United Nations High Commissioner for Refugees (UNHCR) and the International Organization for Migration (IOM), the method of integrating special populations within the framework. This could be done by updating the capacities' indicators to involve displaced populations and providing specific recommendations to States Parties hosting these populations. Moreover, mobilizing financial resources for host countries should be considered.

### Acknowledging risk mapping as a pre-requisite to a successful response

Benjamin Franklin stated in the eighteenth century, "By failing to prepare, you are preparing to fail" [63]. Risk mapping is an essential component of any early-warning system. This study highlighted the importance of risk mapping amid the instabilities of Lebanon. This could be done by brainstorming suspected risks.

### Trengthening research on IHR (2005) implementation in Lebanon

There is a need for academia in Lebanon to invest their efforts in spotting light on IHR. Producing evidence-based research on IHR would serve as an advocacy approach to strengthen political commitment. Moreover, it is essential to produce policy briefs on the importance of the IHR framework in preventing, detecting, and responding to public health emergencies and distributing them to policy-makers. There is a need to conduct research-based studies on each of the 13 capacities implementation in the Lebanese context.

## Conclusion

Lebanon's unique political context has consequences on IHR governance. The alarming level of migration among human resources working with IHR as a result of the current economic crisis is leading to resource depletion that may hamper Lebanon's ability to prevent, detect, and respond to future health emergencies. This magnifies the need for States Parties' support to mobilize financial resources to the IHR pool fund to countries facing similar challenges as Lebanon.

Our study is the first to identify the impact of refugees on the IHR capacities in a country. Although we could not address the effect of refugees' presence on capacities’ scores over the years due to data gaps, the study highlighted that there is a need for WHO and its Member States to mobilize more financial and human resources to support countries hosting displaced populations in maintaining their IHR capacities. The study also highlighted the need to acknowledge the role of refugees in establishing coordination mechanisms between governmental authorities and non-governmental and international organizations, which is advantageous for IHR coordination mechanisms.

The COVID-19 pandemic was a litmus test for Lebanon to invest in promoting governance to establish an accountability framework for all the concerned parties and strengthen IHR compliance. COVID-19 will not be the last public health emergency, and with all the compounded crises Lebanon has been assailed by, there is a considerable need to recognize the IHR framework as a fundamental cornerstone and incorporate all its capacities into the Lebanese health system. As stated by Benjamin Franklin, "By failing to prepare, you are preparing to fail."

### Supplementary Information

**Additional file 1:** **Supplementary Material 1.** Interview guide: Analyzing the environment of International Health Regulations 2005 (IHR) in Lebanon – in a context of displacement, crisis, and refugee waves.

** Additional file 2:** **Supplementary** **Material 2. **Summary of the qualitative analysis.

## Data Availability

The datasets generated and/or analyzed during the current study are not publicly available due to privacy concerns but may be available from the corresponding author upon reasonable request.

## Abbreviations

IHR: The International Health Regulations
WHO: World Health Organization
PoEs: Points of Entry
IPC: Infection prevention and control
SPAR: IHR State Party Self-Assessment Annual Report
JEE: The Joint External Evaluation tool
MOPH: The Ministry of Public Health
HazMat: Medical Hazards Management Team
LAEC: The Lebanese Atomic Energy Commission
EOC: Emergency Operation Center
PTC: Professional Training Center
CSO: Civil Society Organizations
EWARS: Early Warning & Response
GIS: Geographic Information System
CL: Cutaneous Leishmaniasis
SARI: Severe Acute Respiratory Infections
CBRN: Chemical, biological, radio-nuclear, and nuclear
AMR: Antimicrobial Resistance
ISOPOD: Isolation and Infection Control Pods and Shelters
PPEs: Personal Protective Equipments

## Footnotes

1 Rafik Hariri University Hospital.

2 Rafik Hariri University Hospital.
