# Supplementary material for: A closer look at the international health regulations capacities in Lebanon: a mixed method study
Source: BMC Health Serv Res. 2024 Jan 11;24:56. doi: 10.1186/s12913-023-10380-3 (PMC10782771; doi:10.1186/s12913-023-10380-3)
Supplement: Supplementary file 1 — Additional file 1: Supplementary Material 1. Interview guide: Analyzing the environment of International Health Regulations 2005 (IHR) in Lebanon – in a context of displacement, crisis, and refugee waves. [file 12913_2023_10380_MOESM1_ESM.docx]

**Supplementary** **Material 1**

**Interview guide: Analyzing the environment of International Health Regulations 2005 (IHR) in Lebanon – in a context of displacement, crisis, and refugee waves**

**Introduction:**

Lebanon ratified the international health regulations (IHR) 2005 in 2007. And it has been working since then to implement the eight-core capacities and the four core capabilities you have in front of you.

(1) National legislation, policy, and financing;

(2) Coordination and national focal point communications;

(3) Surveillance;

(4) Response;

(5) Preparedness;

(6) Risk communication;

(7) Human resource capacity; and

(8) Laboratory

(9) Points of Entry (PoE)

(10) Zoonotic events

(11) Food safety

(12) Chemical events

(13) Radiation emergencies

**Q1:**What can you tell me about the main milestones that Lebanon executed to strengthen the implementation of the IHR (2005) core capacities since the ratification in 2007?

- Can you identify any national health plans implemented in the public or private sectors?
- Can you identify any national health policies or legislations?

**Q2:**Lebanon has been going through many crises since the ratification of IHR (2005); what can you tell me about the challenges facing IHR implementation in the Lebanese context?

- Could you specify any challenges in responding to the core capacities targets?
- Could you specify any challenges in collecting data?
- Could you specify any challenges in reporting data or communicating findings?

**Q3:**Since 2011, Lebanon has been responding to the influx of Syrian refugees; based on your experience, what barriers do refugees impose on the development of Lebanon’s core capacities?

- National legislation, policy, and financing
- Coordination and national focal point communications
- Surveillance
- Response
- Preparedness
- Risk communication
- Human resource capacity
- Laboratory

**Q4:** Based on your experience, what could be done to implement IHR during the crisis better? Can you give some recommendations?

- National health plans?
- Communication strategies?
- Financial recommendations?
- Technical recommendations?

**Q5:** Are there any lessons to learn from the experience of IHR implementation in Lebanon - in a context of displacement, crisis, and refugee waves?

**Q6:**Are there any additional concerns or issues you would like to talk about? Do you have any questions for us?
