# Supplementary material for: A closer look at the international health regulations capacities in Lebanon: a mixed method study
Source: BMC Health Serv Res. 2024 Jan 11;24:56. doi: 10.1186/s12913-023-10380-3 (PMC10782771; doi:10.1186/s12913-023-10380-3)
Supplement: Supplementary file 2 — Additional file 2: Supplementary Material 2. Summary of the qualitative analysis. [file 12913_2023_10380_MOESM2_ESM.docx]

**Supplementary** **Material 3**

**Summary of the qualitative analysis**

| **Themes** | **Code** | **Sub-code** | **Summary** | **Quotes** |
| --- | --- | --- | --- | --- |
| Challenges facing IHR in the Lebanese context | Resources shortage | Human resources shortage | Human resources deficit was reported by all key informants as a major barrier to conducting IHR activities, mainly due to the alarming migration rate of human resources from Lebanon. | “*My team is migrating… I lost many staff from my team… some migrated to Chili… some to the USA… to France… to Egypt… to the UAE… the team of the Ministry is migrating one after another…”* |
|  |  | Financial resources shortage | Almost all participants reported financial shortages. | “*I think the main challenge to doing anything under IHR is actually the budget”* |
|  |  | Supplies shortage | Three participants agreed on the shortage of the supplies necessary for IHR activities. | *“we took samples only when there was a suffocation case because now there are shortages in staff, finance, and kits. So I prefer to leave the kits to the real threats, not the suspected ones”* |
|  |  | Power shortage | Three participants acknowledged that power shortage imposes barrier on IHR implementation. | *“when there is no electricity, we are not able to perform… if there is no internet, our work will stop”* |

|  | Political challenges | N/A | Six key informants mentioned that the political context in Lebanon is the main challenge. | *“This did not pass. This is, of course, related to the political cleavages and all the context in the country.”* |
| --- | --- | --- | --- | --- |
|  | Governance challenges | N/A | Five key informants believe that the governance of IHR is the main challenge. | *“Each component has its own challenges, actually, but if you look at it overall, it is an issue of governance; it is the way the government deals with IHR.”* |
|  | IHR knowledge gap | N/A | Two of the key informants working outside WHO and MOPH revealed a gap in IHR knowledge in the NGOs sector. | *“There is a gap in the knowledge… not centrally, but more for the staff at the portals…”* |
| IHR strengths | Constant collaboration between some entities | N/A | The collaboration issue among IHR stakeholders was debeatable. Although three key informants asserted a gap in collaboration under IHR, five out of nine respondents reported that several agencies are committed to constant collaboration. | *“What we need to say is that there is coordination between the Ministry of Public Health and other ministries as the Ministry of Agriculture… this is very important for IHR.”* |
|  | High-level capacity | N/A | Four key informants reported that Lebanon has a high level of capacities in terms of experts and laboratory capacities. |  |
|  | IHR awareness | N/A | Two key informants acknowledged the MOPH’s awareness of IHR components as a key strength. | “*The Ministry of public health. All departments are very much aware”* |
|  | Preparedness for the following emergencies | N/A | Two key informants reported that Lebanon's multiple crises and emergencies made the country more and prepared for future emergencies. | *“I can tell you that we will have a more rapid response if another pandemic emerges because we are prepared now”* |
| IHR and refugees | Positive perspective | N/A | Two key informant believe that the Syrian refugees crisis has had a positive impact on IHR implementation. | “*The Syrian crisis itself created a coordination mechanism between the partners … between the government and the UN agencies….these coordination mechanisms helped us to have coordination mechanisms in COVID”* |
|  | Neutral perspective | N/A | Three key informants have neutral perspectives. They believe that refugees' presence does not interfere with the IHR development. | *“They do not interfere in the core capacities development because the surveillance is there. It is expanded automated; the preparedness is there”* |
|  | Negative perspective | N/A | Four key informants stated that the Syrian refugees' crisis increased the prevalence of infectious diseases and the risk of re-introducing eradicated diseases. | “*They suffer from other diseases… the Leishmaniasis cases have increased… other diseases started to be introduced… they have a profile of infectious diseases.”* |
| Recommendations to support the implementation of IHR in the context of Lebanon | Coordination strengthening | N/A | Almost all key informants agreed that multisectoral coordination mechanisms between all concerned entities should be strengthened. | *“ I think the first lesson to learn is that ownership of IHR should be multi-disciplinary, multisectoral, and it should be very high-level decision-making for IHR”* |
|  | Risk communication and communication engagement strategies | N/A | Four key informants asserted that it is essential to RCCE measures, especially those targeted toward the public. | “*…but I think that community engagement and engaging with the community is important…”* |
|  | Resources investment | N/A | Another recommendation reported by four key informants is strengthening the investment in resources, whether financial or human resources. | *“To do mobilizing resources for IHR… do fundraising for IHR to have the attention back to the IHR”* |
|  | Risk mapping | N/A | Risk mapping was mentioned by two key informants as a recommendation to strengthen IHR capacities’ performance. | “*It is important to have risk mapping… this is still not achieved in Lebanon…it is very important to highlight the importance of risk mapping”* |
